# Tailoring Energy Absorption of Curved-Beam Lattices Through a Data-Driven Approach

**DOI:** 10.3390/ma18235377

**Published:** 2025-11-28

**Authors:** Pengting Xiang, Xian Liu, Xiang Chen, Chuang Liu

**Affiliations:** College of Civil Engineering, Nanjing Tech University, Nanjing 211816, China; 202461126002@njtech.edu.cn (P.X.); xianliu_real@163.com (X.L.); 15656461810@163.com (X.C.)

**Keywords:** lattice structure, machine learning, energy absorption, sensitivity analysis

## Abstract

Programmable mechanical metamaterials demonstrate significant potential for realizing high-performance mechanical responses, particularly in the field of energy absorption. In this study, a novel curved-beam thickness gradient lattice structure (CBTGLS) is proposed. Based on an intelligent inverse design framework integrating deep learning and genetic algorithms, the beam thickness and curved-beam control points of the CBTGLS were optimized to maximize its total energy absorption (EA) and specific energy absorption (SEA). Furthermore, this research employed interpretability methods, such as Shapley Additive Explanations (SHAP) and Partial Dependence Plot (PDP), to analyze the influence mechanism of geometric parameters on energy absorption performance, aiming to enhance design efficiency and establish a clear design rationale. The results indicate that the optimized CBTGLS exhibits significant improvements in both EA and SEA. Specifically, compared to a baseline straight-beam lattice structure possessing an identical thickness gradient, SEA of the optimized CBTGLS was enhanced by 49.12%. Among the investigated parameters, beam thickness was identified as having a particularly significant impact on performance. Furthermore, it was observed that a curvature profile bending more towards the outer side of the unit cell is more beneficial for enhancing the energy absorption capabilities of the lattice structure.

## 1. Introduction

Lattice structures constitute a class of structural metamaterials in which periodic or quasi-periodic unit cells serve as the fundamental building blocks. Their mechanical response is governed primarily by architected geometry and topology rather than intrinsic material properties. By tailoring unit cell geometry [1,2,3], connectivity topology [4,5,6], and geometric scale parameters [7,8], lattice architectures can exhibit a wide range of nonconventional mechanical behaviors, including negative Poisson’s ratio [9,10,11], high specific strength [12,13], enhanced energy absorption [14,15,16], tunable stiffness [17], and controllable wave propagation characteristics [18]. This shift from a composition-driven to a geometry-driven design paradigm provides a robust strategy for developing lightweight, high-performance, and multifunctional structures.

Among diverse engineering applications, energy-absorbing lattice structures have garnered considerable attention owing to their critical role in crash safety [19], impact mitigation [20], and lightweight protective systems [21]. Nasim et al. demonstrated that polyamide12 lattice pads can significantly reduce impact-induced head injury through enhanced energy dissipation while maintaining structural rigidity [22]. Acanfora et al. introduced lattice structures into lightweight shock-absorbing devices, demonstrating their capacity to reduce structural weight while simultaneously achieving highly efficient energy absorption [23]. Unlike conventional energy-absorbing materials such as metallic foams [24], which rely predominantly on irreversible plastic deformation, architected lattices are able to dissipate energy through geometry-induced deformation modes, enabling elastic recovery [25,26], stability under repeated loading [27], and high SEA. Therefore, the central challenge is to exploit geometric design variables to achieve a balanced improvement in both lightweight performance and energy absorption efficiency.

To address this challenge, numerous structural design strategies have been proposed. Li et al. proposed an interlocking stitched architecture capable of repeatable and adjustable energy dissipation [28]. Zhang et al. inspired by spinal curvature and turtle carapace morphology, incorporated curved beam elements into the Octet framework to enable thermally tunable mechanical and vibro-isolation responses [29]. Huang et al. developed origami structures featuring curved crease surfaces to regulate quasi-static buckling for controlled energy absorption [30]. In addition, honeycomb structure [31], hierarchical lattices [32], and curved-beam structures [33] have emerged as promising candidates, owing to their high manufacturability, wide parameter tunability, and abundant deformation modes. For example, Feng et al. enhanced the energy dissipation capacity of honeycomb cells by introducing wall curvature [34]; Xu et al. designed sunflower-inspired dual-gradient honeycombs with anisotropic auxetic deformation [35]; Mizzi et al. realized hierarchical reinforced metamaterials exhibiting high specific stiffness and strength while maintaining superior energy absorption [36]. These studies collectively illustrate that energy absorption in lattice systems can be effectively governed through progressive buckling, multi-stage deformation, and hierarchical instability mechanisms. However, their deformation modes and performance tunability remain constrained by limited geometric design degrees of freedom.

Building on these observations, most current lattice designs rely on a limited set of geometric parameters, such as inclination angle [37] and thickness ratio [38]. This low-dimensional parameterization constrains the range of attainable deformation modes and limits the ability to realize controlled multi-stage energy absorption responses. In addition, sudden global buckling or localized collapse frequently induces unstable force–displacement behavior with pronounced stress peaks. Conventional approaches based on parametric finite element sweeps are computationally intensive and often inadequate for exploring complex, high-dimensional design spaces. To overcome the limitations of conventional parameterization methods, such as discontinuous design spaces, limited geometric control, and the high computational cost of large-scale finite element analyses, recent studies have mainly focused on two strategies. The first is the introduction of nonlinear geometric design to achieve continuous structural morphology. The second is the use of intelligent optimization methods, including machine learning [39] and evolutionary algorithms, to improve design efficiency and reduce computational expense. In terms of nonlinear geometric design, Han et al. introduced nonlinearity through simple quadratic arch geometries by shifting strut midpoints; however, such parameterization offers limited control over the curvature distribution [40]. Cho et al. [41] employed Bézier curves to design metamaterials with programmable negative thermal expansion and negative Poisson’s ratio, offering high geometric flexibility. Nevertheless, the structural thickness in their designs is typically uniform or based on a simple bi-material configuration, without exploiting controllable thickness gradients as an additional geometric degree of freedom, which limits the ability to tune buckling sequences and energy-absorption pathways. Regarding intelligent optimization methods, Wei et al. [42] developed a deep learning-based inverse design framework that assembles 11 predefined structural units to achieve programmable stiffness. Although the framework exhibits strong predictive capability, it relies on discrete topological combinations rather than continuous geometric control, and it primarily focuses on linear–elastic stiffness without addressing highly nonlinear behaviors such as energy absorption. Wu et al. [43] constructed a cascaded deep learning and optimization framework to accelerate the programmable mechanics of isotropic lattice structures; however, their geometries are generated through Boolean operations on crystallographic templates, resulting in limited geometric freedom. Song et al. [44] coupled a genetic algorithm directly with finite element analyses to design multi-material vibration–isolation structures, but the absence of surrogate modeling leads to high computational cost, and the use of multiple materials may introduce interfacial failure risks during fabrication. Yu et al. [45] used machine learning to optimize the multi-stable behavior of double-curved beams and introduced variable thickness to regulate mechanical performance. However, the thickness variation was defined through piecewise sections, resulting in discontinuous, step-like geometries that may induce stress concentrations. Moreover, the self-learning cycle used in this work relies on random filtering and has weaker global search capability compared with mature genetic algorithms. Another category of methods based on random perturbation of nodal coordinates greatly expands the parameter space [46], yet the inherent randomness often produces irregular geometries and provides limited insight into the influence of geometric parameters on mechanical responses.

While the aforementioned methods can efficiently identify high-performance designs, existing frameworks mainly focus on target-matching inverse design paradigms rather than global performance maximization, and they do not inherently provide insight into the underlying mechanical mechanisms. To address interpretability, this work incorporates data-driven sensitivity analysis using SHAP [47,48,49] and PDP [50]. These techniques quantitatively elucidate the individual and coupled influence of geometric parameters, such as thickness gradients and Bézier curve control points energy absorption mechanisms. This interpretability ensures physical consistency of the optimized results and provides explicit guidance toward principled design.

Motivated by these considerations, this study proposes a curved-beam thickness gradient lattice structure (CBTGLS) in which curved beam geometries are parameterized via Bézier curves and integrated with a controlled thickness gradient. Bézier parameterization facilitates continuous and precise modulation of curvature profiles, overcoming the discrete and limited tunability inherent to conventional parametrizations. The introduction of thickness gradients induces staged deformation sequences, thereby suppressing abrupt buckling and stabilizing the force–displacement response. Furthermore, a deep learning–genetic algorithm hybrid optimization framework is constructed, where a neural network surrogate model approximates structural response and a genetic algorithm conducts global search within the high-dimensional design space. Finite element simulations, combined with SHAP and PDP analyses, are employed to uncover absorption mechanisms of the proposed architecture. The results offer a transferable reference for the interpretable design of energy-absorbing lattice structures and provide a strategy for enhancing the mechanical efficiency of architected metamaterials.

## 2. Method and Model

### 2.1. Structural Design and Finite Element Analysis

**Figure 1:**
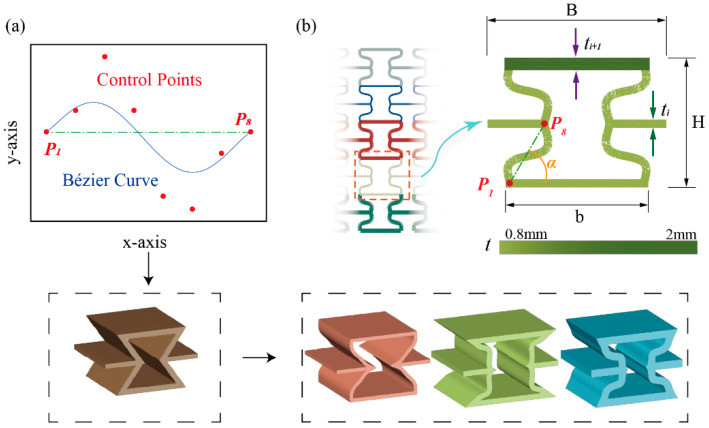
Geometric construction method for CBGLS: (**a**) Diverse unit cell configurations achieved by replacing straight beams on the lateral sides of re-entrant hexagonal lattice structures with curved beams controlled by Bézier curves. (**b**) Geometric parameters of the CBTGLS unit cell.

This study aims to investigate the mechanical properties of a novel re-entrant hexagonal lattice structure (CBTGLS). The core innovation of this design lies in replacing the conventional straight beams in the unit cell with curved beams parameterized by Bézier curves. As shown in Figure 1a, each curved beam is defined by eight control points, forming the geometric configuration of the unit cell. The Bézier curve is calculated according to Equation (1), where the number of control points is 8, and the corresponding parameter *e* is set to 7, since the curve order is determined as one less than the number of control points. The coordinates of these points are precisely specified to ensure a controllable geometric morphology. Specifically, the coordinates of the initial control point are fixed at (0, 0), and the coordinates of the final control point are fixed at (0, $\frac{H/2}{\sin\frac{\pi }{3}}$). The abscissas of the remaining six intermediate control points are sequentially located at equidistant points within the range of [0~$\frac{H/2}{\sin\frac{\pi }{3}}$].

(1) Bq=∑m=0eemqm1−qe−m·Pm, 0≤q≤1

As shown in Figure 1b, *H* represents the distance between the centerlines of the upper and lower horizontal beams of the unit cell; *B* is the width of the unit cell; *b* is the length of the upper and lower horizontal beams; α is the angle between the line connecting the control points in the CBGLS unit cell and the horizontal beams.; and $t_{i}$ is the beam thickness of the unit cells in the i-th layer.

In a single CBTGLS, the curved beams of all unit cells are constructed from the same Bézier curve definition. The beam thickness is identical for all unit cells within the same layer but differs between adjacent layers. This gradient design is achieved by independently controlling the beam thickness of the unit cells in each respective layer.

To systematically investigate the compressive mechanical behavior of the CBTGLS, this study employed the finite element software ABAQUS/Explicit 2023 to generate the dataset. The numerical modeling and computational processes were automated using Python 3.12.9 scripts. The data for each sample included the geometric parameters of the specimen, as well as the corresponding EA, mass (M), and SEA obtained from the finite element simulations. Each specimen was composed of a 5 × 10 array of unit cells, and the material selected was PLA. Given that Sun et al. previously determined the fundamental mechanical parameters and the stress–strain relationship of PLA through quasi-static tensile tests on standard specimens, the material parameters reported in that publication were adopted for the numerical simulations in this work [34]. Table 1 lists the values of the various geometric parameters for the CBTGLS unit cell used in the finite element simulations.

**Figure 2:**
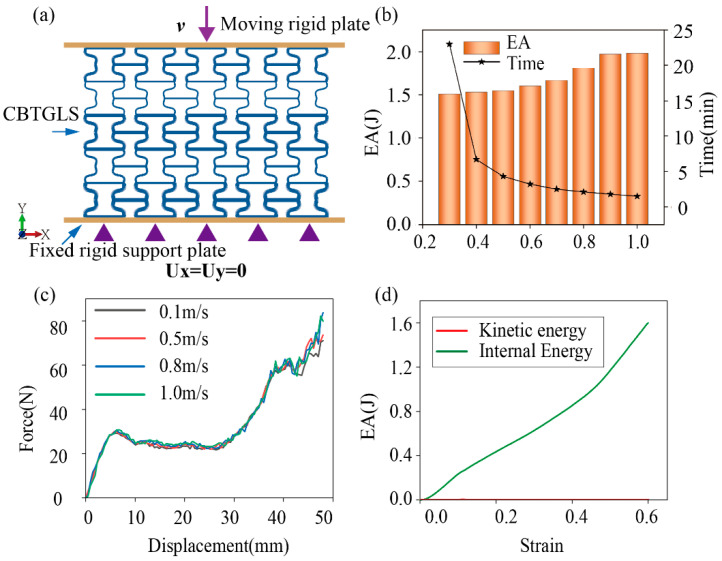
(**a**) Schematic of the finite element simulation for the CBTGLS tensile test. (**b**) Mesh size convergence analysis. (**c**) Velocity convergence analysis. (**d**) Energy relationship produced in the numerical simulation.

The finite element model consists of the CBTGLS specimen, a moving rigid plate, and a fixed rigid support plate. During the simulation process, the support plate remained fully constrained, while the moving rigid plate compressed the honeycomb structure positioned between the two rigid plates at a constant velocity. The compression was applied up to a displacement corresponding to 60% of the honeycomb’s height, as illustrated in Figure 2a. A “General Contact” interaction was employed, with a friction coefficient of 0.2. The numerical simulations were performed using two-dimensional Timoshenko beam elements of type B21.

EA is obtained by integrating the force–displacement curve. Its calculation formula is as follows:

(2) $$\begin{array}{c} EA=\int_{0}^{\delta_{d}}F\;(\delta )\;d\delta \end{array}$$

Here, δ denotes the vertical compression displacement, and F (δ) represents the corresponding compressive force. SEA is calculated as follows:

(3) $$\begin{array}{c} SEA=\frac{EA}{M} \end{array}$$

A convergence analysis was conducted in this study to determine an appropriate element size, ensuring a balance between relative accuracy and computational cost for the data simulations. As shown in Figure 2b, the computational time decreases as the element size increases. It was observed that when the element size is smaller than 0.8 mm, the results approach convergence, while the computational time remains relatively low. Therefore, a mesh size of 0.7 mm was adopted for all finite element models. Additionally, Figure 2c presents the force–displacement curves for loading rates ranging from 0.1 m/s to 1 m/s. It was found that the curve corresponding to a loading speed of 0.8 m/s was nearly identical to the curve at 0.1 m/s. Consequently, to simulate the quasi-static compression behavior of the CBTGLS in a computationally efficient manner, a loading speed of 0.8 m/s was adopted.

By comparing the total energy and kinetic energy of the simulated model in Figure 2d under a compression rate of 0.8 m/s, it is shown that the kinetic energy does not exceed 1% of the total energy, and the hourglass energy is negligible. This confirms the feasibility of performing finite element simulations at a loading rate of 0.8 m/s.

### 2.2. Deep Learning Models and Optimization Algorithms

**Figure 3:**
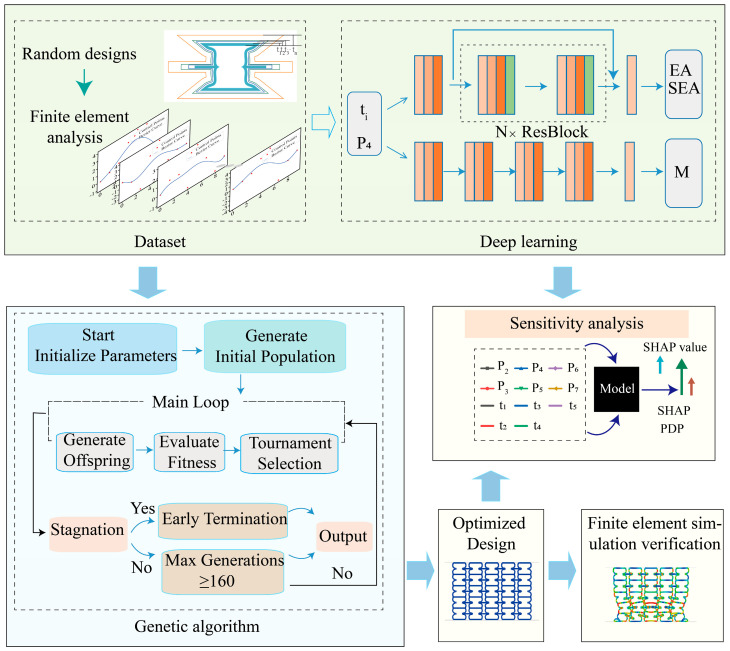
Flowchart of the optimization design for achieving the target performance of lattice structures. Design parameters are randomly sampled to generate candidate lattice structures, which are analyzed in Abaqus to obtain their effective mechanical responses (EA, SEA, and M) for constructing the dataset used to train the surrogate model. The trained model supports both global sensitivity analysis and genetic algorithm-based optimization. The parameters obtained from the genetic algorithm are subsequently re-evaluated through finite element simulations to verify their mechanical performance.

In structural optimization, the objective function often exhibits strong nonlinearity, high coupling, and a multi-modal distribution. Gradient-based methods are prone to convergence at local optima, which hinders the identification of the global optimum. In this study, the design space is high-dimensional, comprising Bézier curve control points $P_{2}$ to $P_{7}$ and thickness gradient parameters for each layer. Moreover, no explicit analytical relationship exists between the geometric configuration and the mechanical response, further increasing the complexity of the optimization problem. GA was employed as the global optimization strategy. EA, SEA, and M are calculated for an initial set of design samples using finite element simulations, which are then used to construct a training dataset for a neural network surrogate model. Based on the trained surrogate model, a global sensitivity analysis was integrated to evaluate the influence of each design parameter, including $P_{2}$ to $P_{7}$ and the layer thickness gradients on the energy absorption performance over the entire design space. By coupling sensitivity analysis with the predictive model, the framework not only enables efficient identification of optimal designs via GA but also provides quantitative insight into the high-dimensional design space. The analysis identifies the parameters with the greatest overall impact, offering critical guidance for structural design and supporting informed decision-making. Figure 3 presents the workflow of the integrated optimization framework for enhancing lattice structure performance.

A finite element analysis approach was employed to generate the dataset required for training the deep learning model. The dataset was subsequently split into training, validation, and test sets with a ratio of 8:1:1. By training the neural network, a mapping between the material parameters and their corresponding mechanical performance was established. During the optimization stage, the trained deep learning model was utilized as the fitness evaluator for the genetic algorithm, leveraging its efficient predictive capability to rapidly compute the fitness values of population individuals. The optimization results were then verified through finite element simulations. If the performance objectives were not met, the iterative process was repeated until the predefined termination criteria were satisfied, ultimately yielding the optimal combination of material parameters that meets the target performance requirements. The mapping relationship between the geometric parameters and the corresponding performance is expressed as follows:

(4) $$G_{predict}=F\left(P_{m},t_{i}\right)$$

Here, $P_{m}$ and $t_{i}$ denote the design parameters, where the subscript m refers to the specified control point, and the subscript i indicates the layer of the unit cell.

The deep learning model consists of three independent multilayer perceptron sub-networks, with prediction targets including the M of the CBTGLS, as well as its EA and SEA generated during compression to 60% of its height. Each sub-network comprises five fully connected hidden layers, as illustrated in Figure 3. Each deep learning model is trained using the Adam optimizer and employs Leaky_ReLU with a slope of 0.1 as the activation function. Batch normalization is incorporated into the models. Furthermore, for the prediction models targeting SEA and EA, residual networks are utilized to prevent gradient explosion, and dropout is added to mitigate overfitting. The number of residual layers *N* for both of these target models is 2. The loss function is defined as the Mean Squared Error between the model output values and the true labels, and is used to quantify the prediction error. To ensure training stability and accuracy, the learning rate is multiplied by a decay factor after every 80 epochs. Table 2 presents the hyperparameters of the models for different prediction targets, together with the resulting RMSE and MAE values, demonstrating that the models provide reliable and accurate predictions.

**Figure 4:**
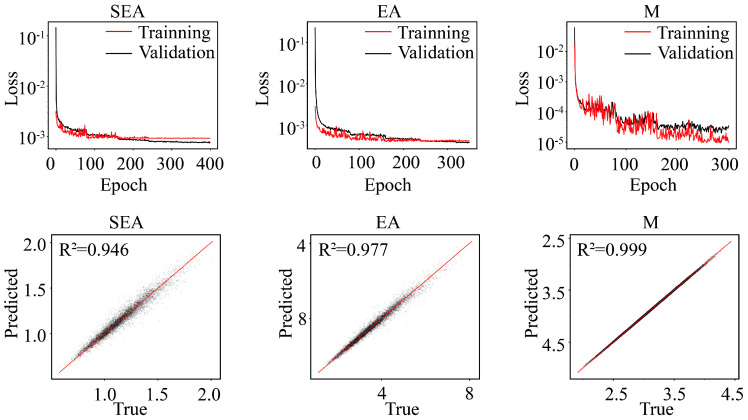
The loss variation curves of the training set and validation set, and comparison between predicted values (DL predictions) and ground truth (FEM results).

The loss variation of each deep learning model during the training process, along with the comparison between the predicted results and the true values, is shown in Figure 4. The loss values for all models remain at a low level, and their corresponding $R^{2}$ values are all close to 1. This sufficiently demonstrates that the predictive capability of each model possesses a high degree of accuracy.

In this study, we investigated the enhancement of EA under a fixed volume and the improvement of SEA for CBTGLS. For different optimization objectives, specific fitness functions were designed, resulting in distinct hyperparameter settings in the genetic algorithm. Table 3 presents the hyperparameter settings of the genetic algorithm for different optimization objectives. The workflow of the genetic algorithm for EA optimization is illustrated in Figure 3. Initially, the algorithm generates an initial population consisting of 10,000 individuals. During the main iterative process, offspring are produced according to the predefined crossover and mutation rates. The fitness of each individual is evaluated using the pretrained M_Net and EA_Net neural network models. The fitness function aims to maximize the predicted EA under strict constraint penalties, while ensuring that the predicted mass approaches the target value. The tournament selection strategy is adopted to select individuals for the next generation. The algorithm terminates when the number of generations reaches 160 or when the optimal solution remains unchanged for 100 consecutive generations, and the best individual is then output. The optimization process for SEA follows the same procedure as that for EA.

## 3. Results and Discussion

### 3.1. Sensitivity Analysis of the Influence of Geometric Parameters on EA

**Figure 5:**
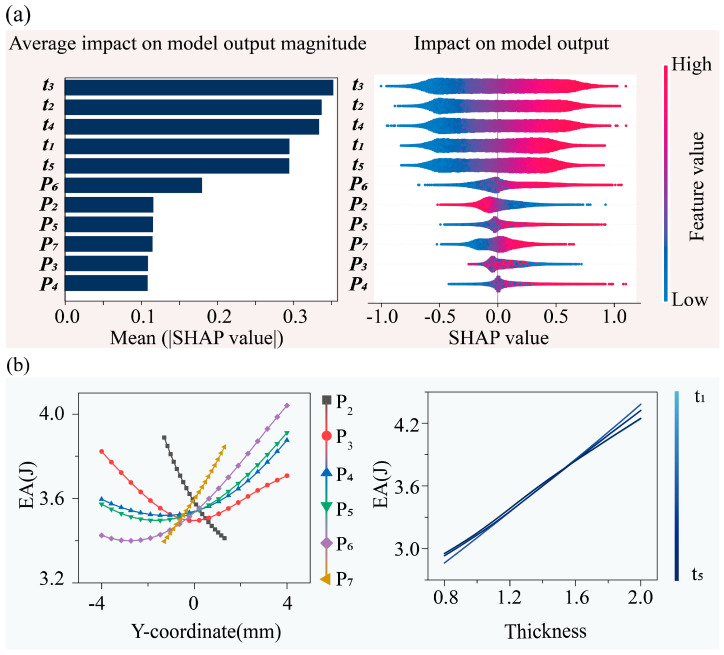
Analysis of geometric parameters’ effects on EA: (**a**) Parameters ranked by mean absolute SHAP values, indicating their overall influence on EA; SHAP summary plot showing the directional effects of parameters and their value relationships: color represents parameter values from low (blue) to high (red), while the *x*-axis (SHAP values) indicates positive or negative effects on EA. (**b**) Dependency analysis results of geometric parameters’ influence on EA based on PDP. Blue lines represent thickness parameters (from light blue to dark blue), while control point parameters are indicated by colored marker shapes.

The SHAP model was employed to analyze the influence of each reference point in the Bézier curve and the beam thickness at different layers on EA and SEA of the CBTGLS under 60% compressive strain. The SHAP values are visualized in Figure 5 to illustrate the effects of different geometric parameters on the energy absorption (EA) of the CBTGLS. In Figure 5a, the left panel presents the mean absolute SHAP values of each parameter, reflecting their overall impact on EA. Among the beam thickness parameters, $t_{3}$, $t_{2}$, and $t_{4}$ exerted the most significant influence on EA, with $t_{3}$ being the most dominant, followed by $t_{2}$ and $t_{4}$, whereas $t_{1}$ and $t_{5}$ exhibited relatively minor and comparable effects. This indicates that beam thickness parameters closer to the middle layer of the lattice structure have a stronger impact on EA. In contrast, the control point parameters exerted weaker overall effects, among which $P_{6}$ was the most prominent, exhibiting the highest mean absolute SHAP value; the remaining control points had similarly low contributions, indicating that their influence on EA is generally lower than that of the beam thickness parameters.

The right panel of Figure 5a further reveals the detailed influence mechanisms of each parameter on EA. The SHAP value distribution of the middle-layer beam thickness $t_{3}$ spans the widest range (−1.0 to 1.0) and exhibits a clear color gradient: high feature values (red) generally correspond to positive SHAP values, whereas low feature values (blue) correspond to negative or near-zero SHAP values, indicating a strong positive effect of $t_{3}$ on EA. Similarly, $t_{2}$ and $t_{4}$ display increasing SHAP values with increasing feature values, although their magnitudes are weaker than that of $t_{3}$. Specifically, $t_{2}$ exhibits a wide distribution with a clear positive trend, while $t_{4}$ ranges from −0.3 to 0.6, falling between $t_{3}$ and $t_{2}$. The influence interval of $t_{1}$ is smaller, showing a positive trend but contributing less overall than the aforementioned parameters. The SHAP distributions of the control points exhibit more complex nonlinear characteristics. Most control points have SHAP values concentrated around zero, confirming their relatively weak influence. Among them, $P_{5}$, $P_{6}$, and $P_{7}$ display positive correlations, where higher feature values correspond to larger SHAP values; conversely, $P_{2}$ shows a negative correlation, with low feature values corresponding to positive SHAP values and high feature values corresponding to negative values. Notably, $P_{3}$ and $P_{4}$ exhibit SHAP values spanning both positive and negative intervals for high and low feature values (manifested as a mixture of red and blue points), indicating that their effects on EA are significantly influenced by interactions with other parameters and display pronounced nonlinearity.

PDP in Figure 5b provides a global interpretation of how individual geometric parameters influence EA. The results reveal that the effects of control points on EA exhibit complex and nonlinear trends. Specifically, EA decreases with the increase in control point $P_{2}$. For control point $P_{3}$, the closer its vertical coordinate is to zero, the lower the corresponding EA; conversely, as the vertical coordinate deviates further from zero, EA increases, showing a distinct nonlinear relationship. Moreover, based on the slope of EA-Y coordinate curves, it can be observed that the closer a control point is to the center of the unit cell, the steeper the slope becomes. This indicates that control points located nearer to the cell center have a stronger positive correlation with EA of the CBTGLS structure. The SHAP analysis of the average influence of geometric parameters further shows that $P_{6}$ has the most significant effect on EA of CBTGLS, rather than $P_{7}$. This is attributed to the wider range of $P_{6}$ compared with $P_{7}$, which leads to a larger fluctuation range in the influence of $P_{6}$ on EA.

As shown in the right panel of Figure 5b, the relationship between the beam thickness of each layer and EA indicates that the effect of layer thickness on EA is predominantly linear, and the trends among different layers are generally consistent. Furthermore, the influence range of beam thickness on EA is broader compared with that of the control points.

In summary, EA of the CBTGLS structure is primarily governed by a positive correlation with beam thickness, and the influence of beam thickness across different layers is comparable. The secondary factor affecting EA is the position of the control points: control points located closer to the center of the unit cell, with larger vertical coordinates, contribute more positively to the enhancement of EA. However, the control point $P_{2}$, located near the lateral boundary of the unit cell, exhibits a negative correlation with EA of CBTGLS.

### 3.2. Sensitivity Analysis of the Effects of Geometric Parameters on SEA

**Figure 6:**
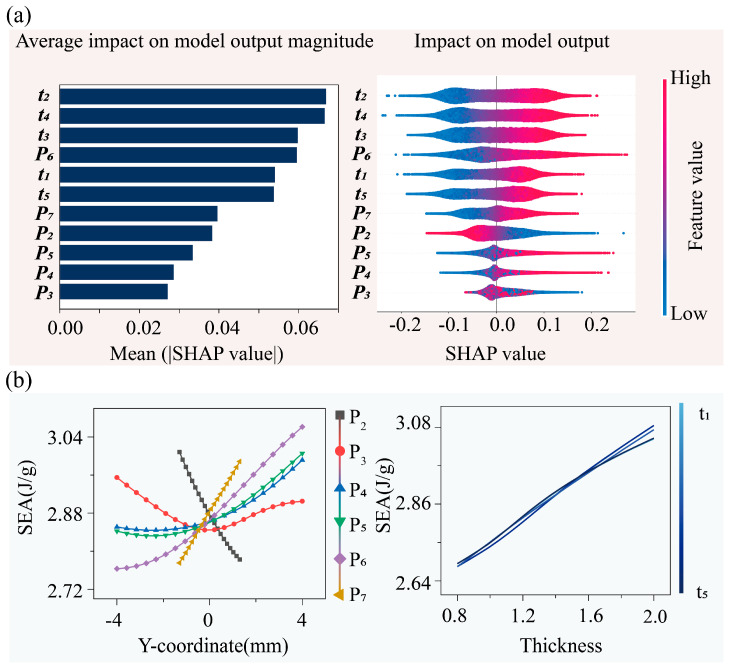
Analysis of geometric parameters’ effects on SEA: (**a**) Parameters ranked by mean absolute SHAP values, indicating their overall influence on SEA. SHAP summary plot showing the directional effects of parameters and their value relationships. (**b**) Dependency analysis results of geometric parameters’ influence on SEA based on PDP. Blue lines represent thickness parameters (from light blue to dark blue), while control point parameters are indicated by colored marker shapes.

The bar chart in the left panel of Figure 6a presents the mean absolute SHAP values of each parameter, reflecting their overall impact on SEA. Among the beam thickness parameters, $t_{2}$ and $t_{4}$ exhibit the strongest average influence on SEA, followed by the middle-layer beam thickness $t_{3}$ and control point $P_{6}$, whereas $t_{1}$ and $t_{5}$ have relatively minor and comparable effects. The remaining control point parameters generally show weak contributions to SEA, with $P_{3}$ and $P_{4}$ exhibiting the smallest average influence.

The SHAP value distribution in the right panel of Figure 6a further elucidates the specific influence mechanisms of each parameter on SEA. The middle-layer beam thickness parameters $t_{2}$ and $t_{4}$ display the widest SHAP value distributions, and the SHAP distributions of beam thickness across different layers show a consistent trend: high feature values (red) generally correspond to positive SHAP values, whereas low feature values (blue) correspond to negative or near-zero values, forming a clear color gradient. This indicates a strong positive effect of beam thickness on SEA. Most control points exhibit SHAP values concentrated around zero, confirming their relatively weak influence. Among them, $P_{5}$, $P_{6}$, and $P_{7}$ display positive correlations, with higher feature values corresponding to larger SHAP values; in contrast, $P_{2}$ shows a negative correlation, where low feature values correspond to positive SHAP values and high feature values correspond to negative values. Notably, for control points $P_{4}$, $P_{5}$, and $P_{6}$, larger feature values are associated with larger SHAP values, exceeding the SHAP ranges of most other geometric parameters, indicating that increasing the vertical coordinates of these control points strongly promotes SEA. Moreover, control points $P_{3}$, $P_{4}$, and $P_{5}$ exhibit SHAP values spanning both positive and negative intervals for high and low feature values (manifested as a mixture of red and blue points), suggesting that their effects on SEA are significantly influenced by interactions with other parameters.

The PDPs in the left panel of Figure 6b provide a global perspective on the influence of individual geometric parameters on the SEA of the CBTGLS. The effects of the control points on SEA exhibit complex and nonlinear trends. Specifically, SEA decreases with increasing values of control point $P_{2}$. For control point $P_{3}$, the closer its vertical coordinate is to zero, the smaller the corresponding SEA, indicating a pronounced nonlinear effect. Except for $P_{2}$, larger vertical coordinates of the remaining control points generally favor an increase in SEA. Moreover, the slopes of SEA-Y coordinate curves suggest that control points located closer to the center of the unit cell have a stronger positive correlation with SEA. In this study, $P_{7}$ does not exert the most significant influence on SEA because its vertical coordinate range is smaller than that of the other control points. The right panel of Figure 6b shows the relationship between beam thickness at different layers and SEA. The influence of beam thickness on SEA is nearly linear across all layers, with similar trends observed for beams in different layers. It is also evident that the influence of beam thickness on SEA is broader than that of the control points. In summary, SEA of the CBTGLS is primarily governed by the positively correlated effects of beam thickness, with similar contributions from beams in different layers. Control points are secondary factors influencing SEA, and their effect is closely related to their position: control points closer to the center of the unit cell, with larger vertical coordinates, contribute more significantly to SEA enhancement, whereas edge-located control points, such as $P_{2}$, exhibit negative correlations, which reduce the SEA performance of the structure.

## 4. Optimized Design

This section discusses the enhancement of the EA and SEA performance of the CBTGLS through an optimization-based design, in which EA improvement is achieved under a prescribed mass constraint. A comparison of SEA is conducted between the optimized CBTGLS and the conventional straight-beam re-entrant hexagonal lattice structure. Furthermore, based on the SHAP interpretable machine learning framework, the mechanisms underlying the improved EA and SEA of the optimized CBTGLS are analyzed, enabling quantitative interpretation of the predictions generated by individual models.

### 4.1. EA Improvement Under Mass Constraints

In this section, the primary objective is to optimize the EA of the CBTGLS under a mass constraint of 3.3 g and to perform a comparative analysis with the EA of CBTGLS specimens in the dataset that have the same mass of 3.3 g. The fitness function employed in this study is consistent with Equation (5), with the optimization goal set to maximize the target function, where the parameter k is assigned a value of 2, and $N_{1}$ is set to 1.

(5) $$f_{1}=-\left(\frac{k}{N_{1}}\sum_{j=1}^{N_{1}}\left|{\hat{m}}_{j}-m_{j}\right|-\bar{EA}\right)$$

The parameter $m_{j}$ represents the mass of the lattice structure constrained to 3.3 g, while ${\hat{m}}_{j}$ denotes the corresponding prediction from the neural network. $\bar{EA}$ represents the average value of the mechanical property to be optimized, and k is the penalty weight determined according to the optimization objective.

**Figure 7:**
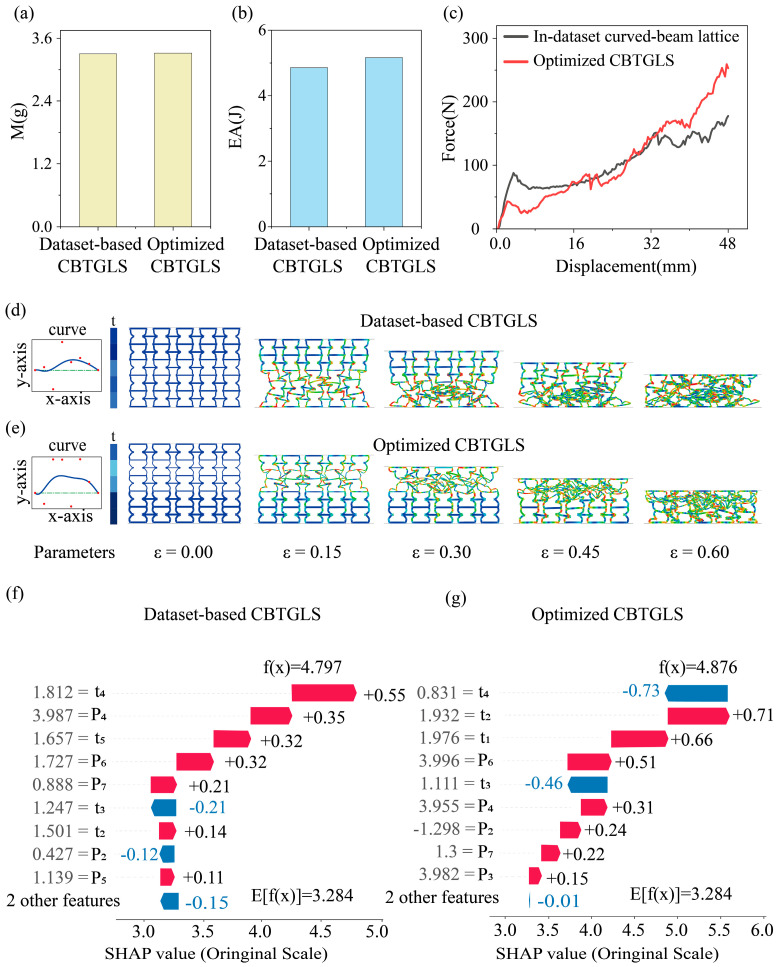
(**a**) Mass comparison chart. (**b**) Energy absorption comparison chart. (**c**) Force–displacement curve comparison chart. (**d**) FEM results of in-dataset CBTGLS. (**e**) FEM results of optimized CBTGLS. (**f**) SHAP waterfall chart of EA for in-dataset CBTGLS. (**g**) SHAP waterfall chart of EA for optimized CBTGLS.

Figure 7a presents a comparison between the mass of the optimized CBTGLS and the target mass from the dataset. The optimized CBTGLS has a mass of 3.33 g, which is close to the target mass in the dataset, indicating precise mass control during the optimization process and effectively avoiding significant mass increases associated with enhancements in energy absorption performance. As shown in Figure 7b, the optimized CBTGLS exhibits improved energy absorption compared to the CBTGLS in the dataset. This result demonstrates that under the same mass and volume constraints, the optimized CBTGLS can absorb energy more efficiently, thereby providing superior cushioning performance in practical applications. The compression force–displacement curves in Figure 7c indicate that during the initial compression stage, the optimized CBTGLS generates lower reaction forces than the dataset CBTGLS. However, as the compression progresses, the optimized CBTGLS sustains significantly higher forces, indicating that the optimized structure can provide more stable load-bearing capacity over a larger deformation range, more effectively resist external impacts, and achieve higher energy absorption and conversion efficiency.

Figure 7d,e depict the thickness gradient distribution and the curvature configuration of the beams for the dataset CBTGLS and the optimized CBTGLS, respectively. In the optimized CBTGLS, the beam thicknesses across different layers of the unit cell exhibit notable variations, with the third and fourth layers being thinner than both other layers and the corresponding beams in the dataset CBTGLS. This feature accounts for the relatively lower reaction force observed during the initial compression stage. Furthermore, the curved beams in the optimized CBTGLS protrude more prominently toward the exterior of the unit cell compared to those in the dataset, with the corresponding control point y-coordinates exceeding those in the dataset. This observation is consistent with the sensitivity analysis, which indicated that increasing the vertical coordinates of control points closer to the center of the unit cell enhances EA of the structure.

Additionally, during the later stages of compression, the optimized CBTGLS maintains a stable increase in reaction force. This behavior is attributed to the bottom two layers of unit cell beams, which undergo the majority of deformation and have been optimized to possess greater thickness. The increased beam thickness effectively enhances the equivalent stiffness (EA) of the structure. This conclusion aligns not only with the sensitivity analysis results but also with the geometric features of the optimized CBTGLS configuration. To gain deeper insight into the influence of different geometric parameters on the energy absorption (EA) of CBTGLS structures, the SHAP method was employed for interpretability analysis. Figure 7f presents the SHAP waterfall plot for the CBTGLS structures within the dataset. By analyzing the SHAP values corresponding to each geometric parameter, the contribution of each parameter to EA can be quantified. The figure shows that the parameter $t_{4}$ exhibits the largest positive contribution to EA, indicating that increasing the beam thickness generally enhances the energy absorption. However, since the third-layer unit cell beams in the dataset CBTGLS are relatively thin, this geometric parameter produces a negative effect on EA for these structures. In addition, $P_{4}$ also has a considerable positive influence, indicating that increasing the vertical coordinate of control points closer to the center layer of the unit cell favors the enhancement of CBTGLS energy absorption. Conversely, $P_{2}$ exerts a negative effect on EA due to its positive value. The combined influence of these parameters allows the dataset structures to achieve an energy absorption level of f (x) =4.797 from a baseline of E[f (x) ]=3.284. Figure 7g shows the SHAP waterfall plot for the optimized CBTGLS structures. Compared to the dataset structures, the optimized structures exhibit a reduced value of $t_{4}$, which still contributes directionally to EA. Meanwhile, the increases in $t_{1}$, $t_{2}$, and $P_{6}$ make them the most significant contributors to the enhancement of EA. Additionally, because $P_{2}$ assumes a negative value in the optimized structure, it further promotes the energy absorption of CBTGLS. These observations indicate that the optimization process effectively reallocated the weights of different parameters to achieve more efficient energy absorption. The output function value of the optimized model, f (x) =4.876, exceeds that of the dataset structures, demonstrating the effectiveness of the optimization.

### 4.2. Design for Maximizing SEA

This section focuses on the enhancement of SEA performance. This is primarily achieved by optimizing the geometric parameters of the CBTGLS to improve its SEA. The corresponding fitness function for the genetic algorithm is as follows:

(6) $$f_{2}=Z$$

The genetic algorithm employed a strategy of maximizing the fitness function; therefore, Z serves as the optimization objective.

**Figure 8:**
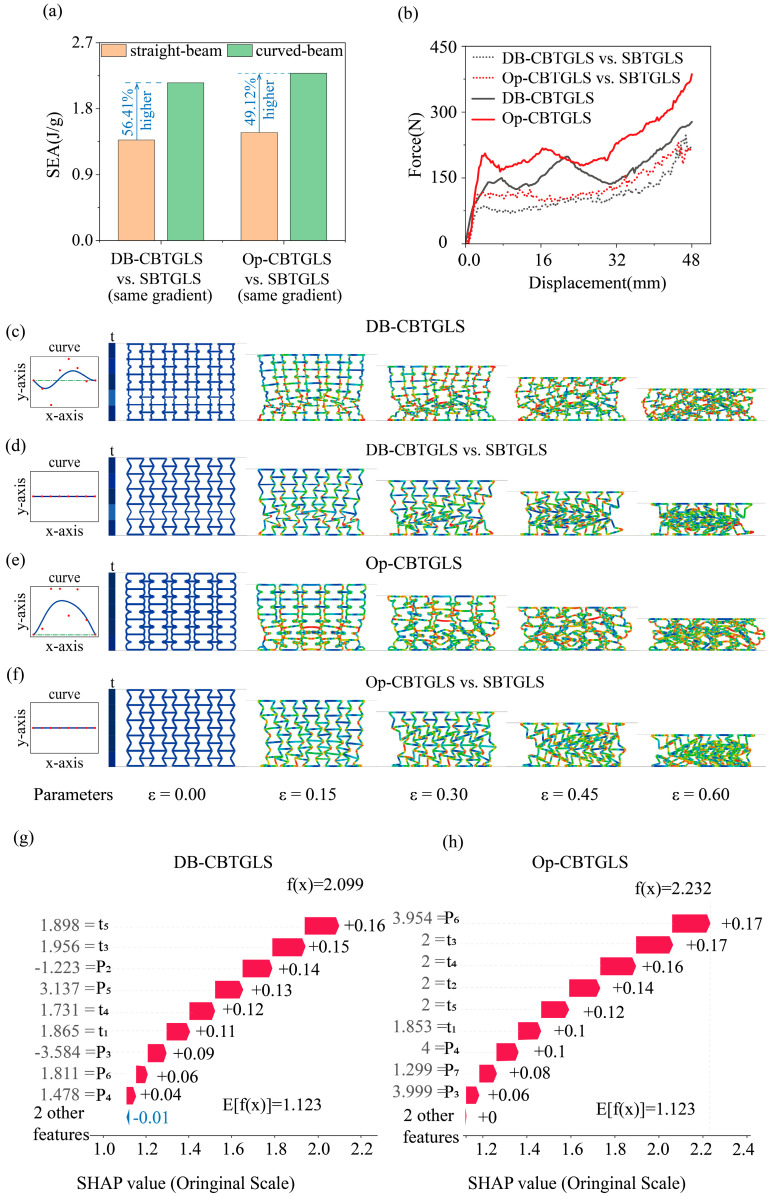
(**a**) SEA comparison chart. (**b**) Force–displacement curve comparison chart. (**c**) FEM of in-dataset CBTGLS. (**d**) FEM of the straight-beam lattice structure with the same gradient as the in-dataset CBTGLS. (**e**) FEM of optimized CBTGLS. (**f**) FEM of the straight-beam lattice structure with the same gradient as the optimized CBTGLS. (**g**) SHAP waterfall chart of in-dataset CBTGLS with optimal SEA. (**h**) SHAP waterfall chart of optimized CBTGLS for SEA.

Figure 8a presents a clear comparison of SEA among three sets of lattice structures using a bar chart. First, the comparison between the CBTGLS with the highest SEA within the dataset (DB-CBTGLS) and the straight-beam lattice structure with the same gradient (SBTGLS) shows that the curved-beam lattice exhibits a 56.41% higher SEA than the straight-beam counterpart. Meanwhile, the optimized CBTGLS demonstrates a 49.12% improvement in SEA compared to the predesigned straight-beam lattice with the same gradient, indicating a significant advantage of the curved-beam design in energy absorption. Moreover, the optimized CBTGLS exhibits a higher SEA performance than the best-performing configuration in the dataset, confirming the effectiveness of the proposed optimization strategy in enhancing SEA. These results indicate that the optimization design can further unlock the latent energy absorption potential of curved-beam lattice structures.

Figure 8b compares the compression force–displacement responses of different lattice structures. The curve of the dataset’s CBTGLS with the highest SEA differs from that of the straight-beam lattice with the same gradient, showing that the curved-beam lattice absorbs more energy over the same displacement range. The compression force–displacement curve of the optimized CBTGLS relative to the straight-beam lattice highlights the advantage of optimization: under large compression, the optimized CBTGLS exhibits higher reaction forces, enabling more efficient energy dissipation under loading and confirming that the curved-beam lattice structure optimized design positively affects SEA. Figure 8c,d illustrate the deformation process of the dataset’s CBTGLS with the highest SEA and the straight-beam lattice with the same gradient during compression. The deformation of CBTGLS shows that, as compression increases, the internal beam elements undergo orderly bending, which facilitates energy absorption and dissipation. In contrast, the straight-beam lattice primarily deforms at the nodes under the same strain, with energy absorbed through nodal bending, which is relatively less efficient. Figure 8e,f present the deformation of the optimized CBTGLS compared with the straight-beam lattice. The optimized CBTGLS exhibits superior deformation behavior at all strain stages; the curved-beam design allows for a more uniform stress distribution and more comprehensive overall deformation, enabling continuous energy absorption over a larger compression range. Analysis of the geometric parameters from Figure 8c,e show that the optimized CBTGLS has thicker beams than the dataset’s CBTGLS, and the optimized curved beams protrude more toward the exterior of the unit cell. Corresponding control points have larger y-coordinates compared with those of the dataset’s lattice. These characteristics are consistent with the sensitivity analysis, which indicated that increased beam thickness and higher control point y-coordinates enhance SEA, validating the accuracy of the sensitivity analysis. The SHAP method was used to interpret the contributions of geometric parameters to SEA for both the dataset’s CBTGLS with the highest SEA and the optimized CBTGLS. Figure 8g presents the SHAP waterfall chart for the dataset’s best-performing CBTGLS, showing the contribution of each geometric parameter to SEA. Among them, parameter $t_{5}$ has the most significant positive contribution, with a SHAP value of 1.898. Parameters $t_{3}$, $P_{2}$, and $P_{5}$ also contribute positively, with SHAP values of 1.956 and 3.137, noting that $P_{2}$ has a negative value. Parameters $t_{2}$ and $P_{7}$ exert slight negative effects, while other parameters have minor contributions. Overall, the predicted SEA f (x) for the dataset’s optimal CBTGLS is 2.099, indicating that the combined effect of all parameters results in superior energy absorption performance. Figure 8h shows the SHAP waterfall chart for the optimized CBTGLS. Compared with the dataset’s structure with the highest SEA, the influence of geometric parameters is significantly altered. The positive contribution of $P_{6}$ is most pronounced, with a SHAP value of 3.954; the y-coordinate of $P_{6}$ is much larger than in the dataset’s CBTGLS, indicating that controlling this point during optimization enhances SEA. Meanwhile, the beam thicknesses of all layers reach the upper limits of the design range. Compared with the CBTGLS samples in the dataset, this increase in thickness enhances the positive contribution of the beams to SEA, which is consistent with the conclusion obtained from the sensitivity analysis that increasing beam thickness improves SEA. This agreement validates the reliability of the sensitivity analysis. In addition, for the optimized CBTGLS, all geometric parameters contribute positively to SEA, further demonstrating the feasibility and effectiveness of the proposed optimization design.

**Figure 9:**
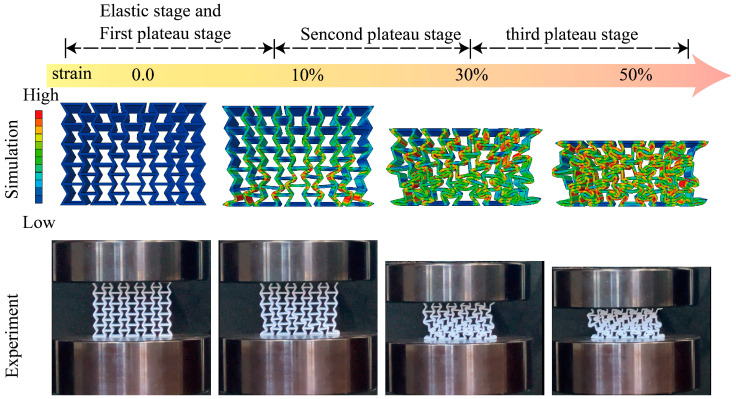
Experimental and simulated deformation comparison of the straight-beam lattice structure under quasi-static compression.

In addition, to further validate the reliability of the numerical model, a compression test was performed on the straight-beam lattice structure specimen. Using the experimentally measured geometry and loading conditions, a corresponding finite element model was established for comparison. The agreement between the simulated stress distribution and the experimentally observed deformation pattern is remarkably high: the locations of beam bending, the evolution of local buckling, and the overall collapse mode predicted by the simulation closely mirror those captured in the experiment. This strong consistency demonstrates that the numerical model accurately reproduces the physical deformation behavior of the lattice structure, thereby confirming the robustness and credibility of the finite element framework employed in this study. The corresponding experimental deformation images and simulated stress–deformation contours are presented in Figure 9, further illustrating their close agreement.

## 5. Conclusions

In this study, curved-beam lattice structures were constructed using Bézier curve–based beam geometry and combined with a thickness gradient design. The resulting CBTGLS exhibit higher energy absorption efficiency under compression compared to conventional straight-beam re-entrant hexagonal lattice structures. By employing a collaborative optimization strategy integrating deep learning and genetic algorithms, the energy absorption performance of CBTGLS under the same volume constraints is significantly enhanced, achieving SEA values that exceed the range of the original dataset. Model interpretability analyses using SHAP and PDP indicate that beam thickness parameters have a pronounced and positive effect on both EA and SEA, particularly for the middle-layer beams. In contrast, the influence of control point parameters is relatively weaker and strongly position-dependent: control points near the center of the unit cell exhibit a significant positive effect on EA and SEA, whereas control point $P_{2}$, located near the unit cell edge, shows a negative correlation, reducing the structure’s EA and SEA. Therefore, in the design of CBTGLS, priority should be given to optimizing the beam thickness in the middle layers and the control points near the unit cell center to maximize energy absorption. These findings provide practical guidelines for designing high-performance curved-beam lattice structures.

## Figures and Tables

**Table 1:** The geometric parameters of CBTGLS.

Parameters (mm)	$P_{2}$	$P_{3}$	$P_{4}$	$P_{5}$	$P_{6}$	$P_{7}$
Range	[−1.3~1.3]	[−4~4]	[−4~4]	[−4~4]	[−4~4]	[−1.3~1.3]
Parameters	B (mm)	b (mm)	H (mm)	α (°)	$t_{i}\;$(mm)	
Value	24	18	16	60	[0.8~2]	

**Table 2:** Parameter settings for each target neural network.

Target Output	Batch-Size	Number of Neurons	Dropout (from Front to Back)	Initial Learning Rate	Epoch	Decay Factor	RMSE	MAE
SEA	400	55	0.13	0.02	400	0.3	0.05	0.04
EA	600	50	0.1	0.02	350	0.3	0.16	0.12
M	2000	50		0.03	300	0.3	0	0

**Table 3:** Hyperparameters of GA for different optimization objectives.

Target	Initial Population Size	Crossover Probability	Mutation Probability	Number of Iterations
SEA optimization	10,000	0.7	0.2	180
M-constrained EA optimization	10,000	0.7	0.4	160

## Data Availability

The raw data supporting the conclusions of this article will be made available by the authors on request.

## References

[1] Zhang X., Yan S., Xie X., Li Y., Wang C., Wen P. (2024). Multi-dimensional hybridized TPMS with high energy absorption capacity. Int. J. Mech. Sci..

[2] Liu K.-J., Liu H.-T., Zhen D. (2024). Mechanical and bandgap properties of 3D bi-material triangle re-entrant honeycomb. Int. J. Mech. Sci..

[3] Zhang X.G., Jiang W., Zhang Y., Han D., Luo C., Zhang X.Y., Hao J., Xie Y.M., Ren X. (2023). Bending performance of 3D re-entrant and hexagonal metamaterials. Thin-Walled Struct..

[4] Dorin P., Khan M., Wang K.W. (2023). Uncovering and experimental realization of multimodal 3D topological metamaterials for low-frequency and multiband elastic wave control. Adv. Sci..

[5] Liu Y., Wang Y., Ren H., Meng Z., Chen X., Li Z., Wang L., Chen W., Wang Y., Du J. (2024). Ultrastiff metamaterials generated through a multilayer strategy and topology optimization. Nat. Commun..

[6] Zhang J., Liu J., Souslov A., Prado M.T.P., Segurado J., Haranczyk M., Christensen J. (2025). Buckle-barrel correspondence based on topological polarization conversion in mechanical metamaterials. Adv. Mater..

[7] Wu Y., Yao H., Li X., Han S. (2025). Metamaterial shaft with a low poisson’s ratio lattice structure for torsional vibration isolation. Mech. Syst. Signal Process..

[8] Nian Y., Wan S., Avcar M., Wang X., Hong R., Yue R., Li M. (2024). Nature-inspired 3D printing-based double-graded aerospace negative poisson’s ratio metastructure: Design, fabrication, investigation, optimization. Compos. Struct..

[9] Fu M., Liu F., Hu L. (2018). A novel category of 3D chiral material with negative poisson’s ratio. Compos. Sci. Technol..

[10] Gao Y., Kang X., Li B. (2025). Programmable mechanical metamaterials with tunable poisson’s ratio and morphable stiffness. Compos. Part B Eng..

[11] Nugroho W.T., Dong Y., Pramanik A., Selvan M.C.P., Zhang Z., Ramakrishna S. (2023). Additive manufacturing of re-entrant structures: Well-tailored structures, unique properties, modelling approaches and real applications. Addit. Manuf..

[12] Tian L., Gu H., Zhang Q., You X., Wang M., Yang J., Dong S. (2023). Multifunctional hierarchical metamaterial for thermal insulation and electromagnetic interference shielding at elevated temperatures. ACS Nano.

[13] Serles P., Yeo J., Haché M., Demingos P.G., Kong J., Kiefer P., Dhulipala S., Kumral B., Jia K., Yang S. (2025). Ultrahigh specific strength by bayesian optimization of carbon nanolattices. Adv. Mater..

[14] Zhang C., Lu F., Wei T., Huang Y., He Y., Zhu Y. (2024). A novel windmill-shaped auxetic structure with energy absorption enhancement. Int. J. Mech. Sci..

[15] Ha N.S., Tran T.T., Lee T.-U., Giustozzi F., Xie Y.M., Xiang X. (2025). Sustainable polycarbonate hierarchical square honeycomb structures for energy absorption. Thin-Walled Struct..

[16] Zhao Y., Chen L., Du B., Liu H., Chen B., Peng S., Guo Y., Chen L., Li W., Fang D. (2019). Bidirectional self-locked energy absorbing system: Design and quasi-static compression properties. Thin-Walled Struct..

[17] Xi K., Jiang X., Zhao D., Chen G., Ma J., Chen Y. (2025). Design and visualization of a hierarchical metamaterial with tunable stiffness. Research.

[18] Ni H., Liu J., Guo L., Zeng T., Pan G. (2024). A novel star-4 honeycomb with the inclined ligaments for enhanced tunability of wave propagation behaviors. Compos. Struct..

[19] Zhang L., Yang D., Li Q., Qiu J. (2024). Accessing quasi-static impact process by 3D-NPR corrugated metamaterials. Int. J. Mech. Sci..

[20] Yang H., Zhang J., Wang J., Hu J., Wu Z., Pan F., Wu J. (2025). Delocalized deformation enhanced reusable energy absorption metamaterials based on bistable tensegrity. Adv. Funct. Mater..

[21] Sood M., Wu C.-M. (2023). Influence of structural arrangements on static and dynamic properties of additively manufactured polyester elastomer lattice metamaterials. Appl. Mater. Today.

[22] Nasim M., Hasan M.J., Galvanetto U. (2022). Impact behavior of energy absorbing helmet liners with PA12 lattice structures: A computational study. Int. J. Mech. Sci..

[23] Acanfora V., Saputo S., Russo A., Riccio A. (2021). A feasibility study on additive manufactured hybrid metal/composite shock absorbers. Compos. Struct..

[24] Rubino V., Deshpande V.S., Fleck N.A. (2008). The collapse response of sandwich beams with a Y-frame core subjected to distributed and local loading. Int. J. Mech. Sci..

[25] Chen S., Lian X., Zhu S., Li M., Wang B., Wu L. (2023). A re-usable negative stiffness mechanical metamaterial composed of bi-material systems for high energy dissipation and shock isolation. Compos. Struct..

[26] Raj R., Jiyalal Prajapati M., Tsai J.-T., Kumar A., Jeng J.-Y. (2024). Design and additive manufacturing of novel hybrid lattice metamaterial for enhanced energy absorption and structural stability. Mater. Des..

[27] Ravanbod S., Rahmani K., Karmel S., Pande I., Amel H., Branfoot C., Shahidi A.M., Alderson A., Bodaghi M. (2025). From coral to control: Bio-inspired, 3D-printable metamaterials with tuneable quasi-zero stiffness and multi-functional bio-composites. Mater. Des..

[28] Li J., Sui C., Sang Y., Zhou Y., Zang Z., Zhao Y., He X., Wang C. (2024). A flexible, reusable and adjustable high-performance energy absorption system inspired by interlocking suture structures. Int. J. Solids Struct..

[29] Zhang X., Han Y., Zhu M., Chu Y., Li W., Zhang Y., Zhang Y., Luo J., Tao R., Qi J. (2024). Bio-inspired 4D printed intelligent lattice metamaterials with tunable mechanical property. Int. J. Mech. Sci..

[30] Huang J., Lin J., Huang L., Liu Y., Xiang X., Liang Y. (2025). Quasi-static mechanical behaviors of arc curved crease origami metamaterials. Int. J. Mech. Sci..

[31] Li X., Li Z., Guo Z., Mo Z., Li J. (2023). A novel star-shaped honeycomb with enhanced energy absorption. Compos. Struct..

[32] Li R., Ma W.W.S., Niu T., Liu H., Ding J., Song X. (2025). Stiffness and strength enhancement of hierarchical TPMS-based shell lattices via inter-level conformal design. Addit. Manuf..

[33] Han D., Li P., Li P., Li L., Bai C., Fan H., Wang P., Song Z., Yang F. (2025). Utilizing partially-curved-beam to improve stress response and energy absorption performance of auxetic lattice metamaterials. Thin-Walled Struct..

[34] Feng G., Li S., Xiao L., Song W. (2021). Energy absorption performance of honeycombs with curved cell walls under quasi-static compression. Int. J. Mech. Sci..

[35] Xu Y., Huang Y., Yan H., Gu Z., Zhao T., Zhang R., Pan B., Dong L., Liu M., Jiang L. (2023). Sunflower-pith-inspired anisotropic auxetic mechanics from dual-gradient cellular structures. Matter.

[36] Mizzi L., Dudek K.K., Frassineti A., Spaggiari A., Ulliac G., Kadic M. (2025). Lightweight 3D hierarchical metamaterial microlattices. Adv. Sci..

[37] Song K., Li D., Liu T., Zhang C., Min Xie Y., Liao W. (2022). Crystal-twinning inspired lattice metamaterial for high stiffness, strength, and toughness. Mater. Des..

[38] Yang C., Nan Z., Huo Y., Yang Y., Xu P., Xiao Y., Fang Y., Meng K. (2025). Design, characterisation, and crushing performance of hexagonal-quadrilateral lattice-filled steel/CFRP hybrid structures. Compos. Part B Eng..

[39] Zhu C., Bamidele E.A., Shen X., Zhu G., Li B. (2024). Machine learning aided design and optimization of thermal metamaterials. Chem. Rev..

[40] Han S., Ma N., Zheng H., Han Q., Li C. (2024). Intelligently optimized arch-honeycomb metamaterial with superior bandgap and impact mitigation capacity. Compos. Part A Appl. Sci. Manuf..

[41] Cho M.W., Ko K., Mohammadhosseinzadeh M., Kim J.H., Park D.Y., Shin D.S., Park S.M. (2024). Inverse design of bézier curve-based mechanical metamaterials with programmable negative thermal expansion and negative poisson’s ratio via a data augmented deep autoencoder. Mater. Horiz..

[42] Wei Z., Wei K., Yang X. (2024). Inverse design of irregular architected materials with programmable stiffness based on deep learning. Compos. Struct..

[43] Wu D., Xu Z., Guo D. (2025). Machine learning accelerates programmable mechanics in isotropic diamond plate lattices. Int. J. Mech. Sci..

[44] Song X., Yan S., Wang Y., Zhang H., Xue J., Liu T., Tian X., Wu L., Jiang H., Li D. (2025). Genetic algorithm-enabled mechanical metamaterials for vibration isolation with different payloads. J. Materiomics.

[45] Yu J., Shi X., Feng Y., Chang J., Liu J., Xi H., Huang S., Zhang W. (2023). Machine learning-based design and optimization of double curved beams for multi-stable honeycomb structures. Extreme Mech. Lett..

[46] Li X., Qin Y., Sun L., Guo X. (2025). Multimaterial metamaterial inverse design via machine learning for tailorable and reusable energy absorption. ACS Appl. Mater. Interfaces.

[47] Khoshgoftar M.J., Barkhordari A. (2022). Sensitivity analysis and study of parameters affecting auxetic cells with reentrant cell structure. Mater. Today Commun..

[48] Xiang Y., Hou J., Chen X., Tang K., Wang X. (2025). Decoupled design of hybrid mechanical metamaterials via ensembled deep learning. Int. J. Mech. Sci..

[49] Oladipo B., Matos H., Krishnan N.M.A., Das S. (2023). Integrating experiments, finite element analysis, and interpretable machine learning to evaluate the auxetic response of 3D printed re-entrant metamaterials. J. Mater. Res. Technol..

[50] Thawon I., Suttakul P., Wanison R., Mona Y., Tippayawong K.Y., Tippayawong N. (2025). Integrating explainable artificial intelligence in machine learning models to enhance the interpretation of elastic behaviors in three-dimensional-printed triangular lattice plates. Eng. Appl. Artif. Intell..

